# Spinal Cord Transection-Induced Allodynia in Rats – Behavioral, Physiopathological and Pharmacological Characterization

**DOI:** 10.1371/journal.pone.0102027

**Published:** 2014-07-14

**Authors:** Saïd M'Dahoma, Sylvie Bourgoin, Valérie Kayser, Sandrine Barthélémy, Caroline Chevarin, Farah Chali, Didier Orsal, Michel Hamon

**Affiliations:** 1 Centre de Psychiatrie et Neurosciences, Institut National de la Santé et de la Recherche Médicale, INSERM U894, Université Paris Descartes, Paris, France; 2 Neuropsychopharmacologie, Faculté de Médecine Pierre et Marie Curie, site Pitié-Salpêtrière, Paris, France; 3 Laboratoire de Neurobiologie des Signaux Intercellulaires, Centre National de la Recherche Scientifique, CNRS UMR 7101, Université Pierre et Marie Curie, Paris, France; Toronto Western Hospital, Canada

## Abstract

In humans, spinal cord lesions induce not only major motor and neurovegetative deficits but also severe neuropathic pain which is mostly resistant to classical analgesics. Better treatments can be expected from precise characterization of underlying physiopathological mechanisms. This led us to thoroughly investigate (i) mechanical and thermal sensory alterations, (ii) responses to acute treatments with drugs having patent or potential anti-allodynic properties and (iii) the spinal/ganglion expression of transcripts encoding markers of neuronal injury, microglia and astrocyte activation in rats that underwent complete spinal cord transection (SCT). SCT was performed at thoracic T8–T9 level under deep isoflurane anaesthesia, and SCT rats were examined for up to two months post surgery. SCT induced a marked hyper-reflexia at hindpaws and strong mechanical and cold allodynia in a limited (6 cm^2^) cutaneous territory just rostral to the lesion site. At this level, pressure threshold value to trigger nocifensive reactions to locally applied von Frey filaments was 100-fold lower in SCT- versus sham-operated rats. A marked up-regulation of mRNAs encoding ATF3 (neuronal injury) and glial activation markers (OX-42, GFAP, P2×4, P2×7, TLR4) was observed in spinal cord and/or dorsal root ganglia at T6-T11 levels from day 2 up to day 60 post surgery. Transcripts encoding the proinflammatory cytokines IL-1β, IL-6 and TNF-α were also markedly but differentially up-regulated at T6–T11 levels in SCT rats. Acute treatment with ketamine (50 mg/kg i.p.), morphine (3–10 mg/kg s.c.) and tapentadol (10–20 mg/kg i.p.) significantly increased pressure threshold to trigger nocifensive reaction in the von Frey filaments test, whereas amitriptyline, pregabalin, gabapentin and clonazepam were ineffective. Because *all* SCT rats developed *long lasting, reproducible and stable* allodynia, which could be alleviated by drugs effective in humans, thoracic cord transection might be a reliable model for testing innovative therapies aimed at reducing spinal cord lesion-induced central neuropathic pain.

## Introduction

Spinal cord injury (SCI) is a debilitating state which causes not only severe motor dysfunctions, loss of bladder control and impairment of sexual function, but also chronic pain, especially neuropathic pain [1], [2]. Pain can be so severe that some SCI patients would be ready to privilege pain relief at the expense of further deficits in bladder control or sexual function. SCI-induced central neuropathic pain can be localized above-, at- or below- the level of injury and is mostly characterized by allodynia refractory to conventional treatments [2], [3].

Several animal models of SCI-induced neuropathic pain have been developed (through spinal cord contusion, compression, ischemia, section; see [4]), each of them displaying different characteristics in terms of localization, duration, type of pain and even responses to drugs. Although some studies did provide relevant data regarding treatment efficacy and underlying molecular mechanisms [5]–[7], they focused mostly on pain below the lesion produced by contusion or clip compression of the spinal cord. Yet, despite the fact that these SCI models reproduce adequately some types of spinal cord injuries seen in humans, they suffer from limitations because of unavoidable, large, interindividual variations in the extent and severity of evoked lesions [8], [9]. Furthermore, lesion-induced neuroinflammatory processes could be highly variable among SCI rats which underwent the very same lesion procedure [10], so that characterization of actual physiopathological mechanisms underlying neuropathic pain might be a real challenge in, at least, some SCI models.

In contrast to these models, complete transection of the spinal cord would be cleared of such limitations due to unavoidable interindividual variations in the extent and severity of the lesion. Indeed, spinal cord transection (SCT) has already been widely used to study the mechanisms of subsequent locomotor recovery [11]–[13] and reorganization of the somatosensory system [14]–[15] in medullary lesioned rats. However, to date, only few studies showed that the SCT model could be used to investigate spinal lesion-induced neuropathic pain [16], and, indeed, some authors even reported that no neuropathic pain develops in rats with complete SCT [17], [18].

These discrepant data led us to reinvestigate whether or not the rat model consisting of complete SCT at the thoracic level could be a relevant model of central neuropathic pain, allowing studies of underlying physiopathological mechanisms and responses to drugs with patent or potential alleviating properties. Nocifensive responses to mechanical and thermal stimulations were assessed using the validated von Frey filaments test and the paw immersion and acetone drop tests, respectively. We then investigated whether responses to these tests could be affected by acute treatments with various drugs (opioids, antidepressants, anticonvulsants and others) known to alleviate neuropathic pain in SCI patients. Finally, we analyzed by real time quantitative RT-PCR, at different times after thoracic cord transection, the expression of mRNAs encoding proteins implicated in neuroinflammation and neuroplasticity, with particular focus on markers of microglia and astrocyte activation, pro- and anti-inflammatory cytokines (Interleukins IL-1β, IL-6 and IL-10, Tumor Necrosis Factor alpha,TNF-α), Brain-Derived Neurotrophic Factor (BDNF) and nociceptive signaling pathways in dorsal root ganglia (DRG) and spinal cord tissues, for comparison with previous studies aimed at unveiling physiopathological mechanisms associated with neuropathic pain in other SCI models.

## Materials and Methods

### Animals and Ethics Statements

Male Sprague–Dawley rats, weighing 225–250 g (7–8 weeks old) on arrival in the laboratory, were purchased from Janvier Breeding Center (53940 Le Genest Saint Isle, France). They were housed under standard controlled environmental conditions (22±1°C, 60% relative humidity, 12∶12 h light–dark cycle, lights on at 7:00 am), on ground corn cobs (GM-12, SAFE, 89290, Augy, France), with complete diet for rats/mice/hamster (105, SAFE, 89290, Augy) and tap water available *ad libitum*. Before surgery, rats were housed 5 per cage (40×40 cm, 20 cm high) and allowed to habituate to the housing facilities without any handling for at least 1 week before being used. After surgery, all efforts were made to minimize suffering. In particular, SCT rats were housed under the very same conditions, except that each cage was for only two operated rats, so as to avoid as much as possible allodynic contacts between them. All animals were thoroughly examined each day, and in case of any sign of abnormal physiological alterations or suffering appeared, they were immediately sacrificed by a lethal dose of pentobarbital (150 mg/kg i.p.), strictly following the recommendations of the Ethical Committee of the French Ministry of Research and High Education (articles R.214–124, R.214–125). Both the Ethics and Scientific Committee of the French *Institut pour la Recherche sur la Moelle Epinière et l'Encéphale* (IRME; contract to M.H., 2010–2011) and the national (French) Committee for Animal Care and Use for Scientific Research (registration nb.01296.01; official authorization B75-116 to M.H., 31 December 2012) specifically approved the study.

In addition, the Ethical Guidelines of the Committee for Research and Ethical Issues of the International Association for the Study of Pain [19] and the Institutional Guidelines in compliance with French and international laws and policies (Council directive 87–848, October 19, 1987, *Ministère de l'Agriculture et de la Forêt, Service vétérinaire de la santé et de la protection animale*, permissions nb A752128 to S.M., 006228 to S.B., nb 00482 to V.K.) were strictly followed.

### Spinal Cord Transection

Animals underwent surgery under deep isoflurane anaesthesia (3%). Paravertebral muscles were cut bilaterally and the T8 vertebra was opened using a gouge-forceps. Local anaesthesia was made by cooling the spinal cord with cryoflurane (Promedica, France) a few seconds before the lesion. Complete transverse section with ophthalmic scissors at the T8–T9 spinal cord segments level was performed following the procedure described by Antri et al. [11], then sterile absorbable haemostatic gel foam (Surgicel; Ethicon, Somerville, NJ, USA) was inserted into the lesion. Sham-operated animals underwent laminectomy only. At the last step of surgery, muscles were sutured and the skin was closed up by skin clips. Both SCT and sham-operated rats then received antibiotic treatments to prevent staphylococcic infection (oxacillin, Bristol Myers Squibb S.P.A., Italy, 0.3 mg/100 g s.c. once a day during 7 days) and urinary infection (gentamicin, Panpharma, France, 0.2 mg/100 g s.c., immediately after the surgery). No further treatment was administered to operated animals, to avoid potential interference with the development of allodynia and hyperalgesia. For recovery, SCT and sham rats were housed two per cage. The bladder of SCT rats was emptied manually once daily until reappearance of the voiding reflex (usually before the 10^th^ day post surgery) (see Results).

### Tests with Von Frey Filaments

#### Assessment of At-Level Mechanical Allodynia

For assessment of SCT-induced neuropathic-like pain in the cutaneous territory bordering surgery scar, rats were placed individually into a plastic cage (42×24×15 cm) and allowed to adapt to this environment for 1 hour before any stimulation. Tactile allodynia was then looked for with a graded series of von Frey filaments (Bioseb, 92370 Chaville, France) producing a bending force ranging between 0.008 g and 100 g. The threshold pressure to trigger a response (see below) was determined using the “up-down” method [20]. The stimuli were applied 3 times (3 seconds apart) for each filament, within a cutaneous territory of about 6 cm^2^ just rostral to the lesion (see Results). When positive nociceptive behaviors, consisting of either a shake, an attack (filament biting), or an escape reaction [5], [20], occurred, the next lower pressure-von Frey filaments were tested down to the filament producing no response. Then, the next higher pressure-filaments were applied back to the one triggering a response. The minimal force filament causing at least one of these responses (usually biting) allowed determination of the mechanical pressure threshold value. The 100 g filament, chosen as cut-off to prevent tissue injury, induced no nociceptive behavior in the majority (>90%) of naïve rats. To avoid nonspecific responses, only these “non-reactive” rats were selected for surgery and included in the study.

#### Assessment of Mechanical Sensitivity in Body Territories Outside the Allodynic Area

**Figure 1:**
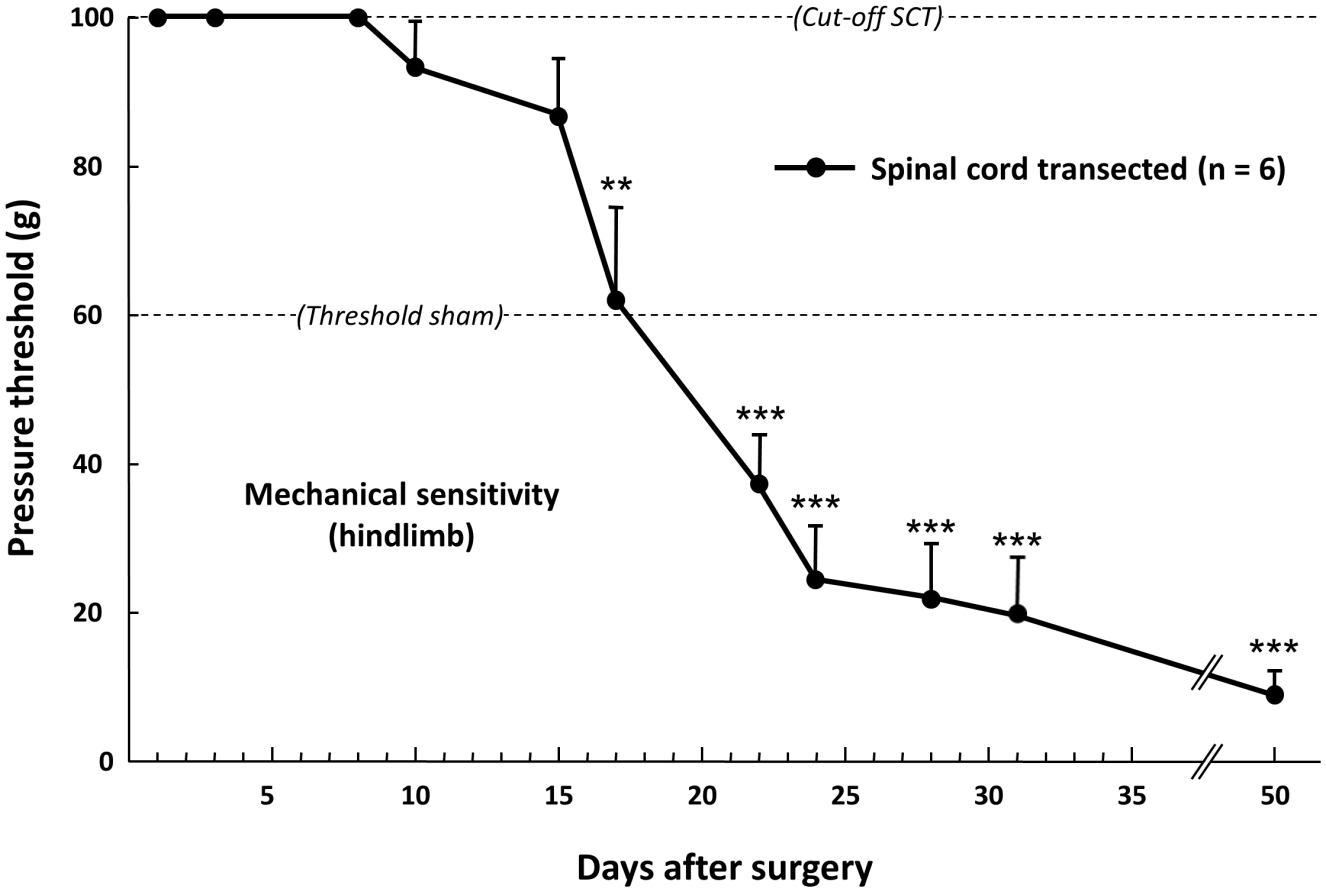
Time-course changes in the pressure threshold value to trigger hindpaw withdrawal in spinal cord-transected rats. Pressure threshold values were determined using a graded series of von Frey filaments applied onto hindpaw. Each point is the mean + S.E.M. of independent determinations in 6 rats. « Cut-off SCT » corresponded to the maximal pressure tested in spinal cord-transected rats; even at this high pressure level (100 g), no response of hindpaws was evoked for the first 9 days post-surgery. The « Threshold sham » corresponded to the minimal pressure (60 g) to which sham-operated animals start to respond by hindpaw withdrawal. ** P<0.01, *** P<0.001, significantly different from 100 g « cut-off SCT » value. One-way ANOVA for repeated measures followed by Dunnett's test.

SCT and sham-operated rats were also subjected to mechanical stimulation with von Frey filaments to assess evoked responses at the level of forepaws, hindpaws, vibrissae pad and other body territories outside the allodynic 6 cm^2^ area just rostral to the surgery scar. For these tests, each rat was placed on a wire grid platform (5×5 mm mesh) under a small plastic (35×20×15 cm) cage for 2 hours, and mechanical sensitivity was determined with a graded series of 9 von Frey filaments (bending force of 4, 6, 8, 10, 12, 15, 26, 60 and 100 g). The “up-down” method [20] was also used at all of these sites. At paw level, stimuli were applied onto the lateral plantar surface of the right forepaw or hindpaw 3 times (3 seconds apart) for each filament. The minimal force filament for which animals presented either a brisk paw withdrawal and/or an escape attempt allowed determination of the mechanical pressure threshold [21]. Usually, the mechanical pressure threshold value to trigger a (non nocifensive) response in naïve healthy rats was around 60 g. Because SCT rats presented large time-dependent changes in mechanical sensitivity (see Fig. 1), higher pressures were also tested, with cut-off fixed at 100 g to avoid any tissue injury.

### Assessment of Thermal Sensitivity

#### Paw Immersion Test

Because variations in skin temperature can affect the responses in nociceptive tests [22], we systematically performed control experiments that consisted of measuring hindpaw skin temperature just before the paw immersion test. The rat was left in its cage and a thermistor probe (Thermocouple thermometer Digi-Sense, Model N°8528-10; Cole-Parmer Instrument Company, Chicago, IL; 15 mm in diameter) was applied onto the plantar surface of hindpaw. Stable temperature readings were obtained after 10 sec with a precision measure of 0.1°C [23].

Thermal sensitivity at the hindpaw level was determined using the paw immersion test in both SCT and sham-operated rats [24]. Briefly, the right hindpaw was immersed into a water bath maintained at 46°C (Polystat, Bioblock Scientific, Illkirch-Graffenstaden, France) for heat stimulation or at 10°C (Ministat, Bioblock Scientific) for cold stimulation, and the latency to struggle reaction (paw withdrawal) was measured to the nearest 0.1 sec.

#### At-Level Cold Allodynia

Cold allodynia in SCT rats was assessed at day 15 post-surgery using a procedure slightly adapted from the acetone drop test described by Baastrup et al. [5]. Four drops of 10 µL acetone were gently deposited within two seconds all around the surgery scar in SCT and sham-operated rats. The number of trunk shakes and the time spent in escape or licking behavior were determined for one min after acetone drops application.

### Pharmacological Treatments

Gabapentin and pregabalin were purchased from Sequoia (Pangbourne, UK). Amitriptyline, baclofen, ketamine and 8-OH-DPAT [(±)-8 hydroxy-2-dipropylamino-tetralin] were from Sigma-Aldrich (Saint-Quentin Fallavier, France). Other compounds were clonazepam (Roche, Basel, Switzerland), cyclotraxin B (BIO S&T, Montreal, Canada), morphine (Pharmacie Centrale des Hôpitaux de Paris, France), tapentadol (Grünenthal, Aachen, Germany), naratriptan and ondansetron (Glaxo Wellcome, Harlow, UK).

All treatments were administered between 2 pm and 4 pm. Routes of administration and doses (as free bases; see Table 1) were chosen according to previous data in the literature (see appropriate references in sections of Results and Discussion). All drugs were dissolved in saline (0.9% NaCl) except baclofen which was dissolved in dimethyl-sulfoxide (DMSO):0.9% NaCl (50∶50) and clonazepam in ethanol:water (50∶50). Drugs or their vehicles were injected acutely 30 days after thoracic cord transection, when mechanical allodynia had fully developed in the 6 cm^2^ area just rostral to the lesion (see Results). For intrathecal injections (of ondansetron), rats were briefly anaesthetized with isoflurane (3% in air), and the needle (26 G) was inserted into the lumbar space between the L5 and L6 vertebrae [25] for administration of the appropriate dose in 20 µL of saline. Von Frey filaments test was then applied (by a skilled experimenter blind to treatments) at various times after acute drugs administration to determine the time course of drug-induced changes in pressure threshold value to trigger nocifensive response (biting of the filament, see Results), until the drug effect completely disappeared. In all experiments, only one treatment was administered per rat.

**Table 1:** Pharmacological treatments tested for potential anti-allodynic effects in spinal cord-transected rats.

Drugs	Pharmacological effect	Dose	Efficacy on biting behavior
Morphine	Opioid receptor agonist	1, 3, 10 mg/kg s.c.	+++
Tapentadol	Opioid receptor agonist and noradrenaline reuptake inhibitor	10, 20 mg/kg i.p.	+++
Ketamine	NMDA receptor antagonist	50 mg/kg i.p.	++
Baclofen	GABA B receptor agonist	10 mg/kg i.p.	+
Clonazepam	Benzodiazepine (agonist)	0.25, 2 mg/kg i.p.	-
Gabapentin	Blockade of calcium channel α2δ subunit	30, 100, 300 mg/kg i.p.	-
Pregabalin	Blockade of calcium channel α2δ subunit	30 mg/kg i.p.	-
Amitriptyline	Tricyclic antidepressant	10 mg/kg i.p.	-
Amitriptyline + Gabapentin	Tricyclic antidepressant + Blockade of calcium channel α2δ subunit	10 mg/kg i.p. +100 mg/kg i.p.	-
Cyclotraxin B	TrkB receptor blocker	20 mg/kg i.p.	-
Naratriptan	5-HT_1B/D_ receptor agonist	0.1 mg/kg i.p.	-
Ondansetron	5-HT_3_ receptor antagonist	20 µg i.t.	-
8-OH-DPAT	5-HT_1A/7_ receptor agonist	0.25 mg/kg i.p.	-

+++: potent anti-allodynic effect (complete recovery of control mechanical sensitivity);

++: potent but short lasting anti-allodynic effect;

+: modest but significant anti-allodynic effect; -: inactive treatment.

### Real Time Quantitative RT-PCR Measurements

SCT- and sham-operated rats were decapitated at various times, from 2 to 60 days, after surgery. DRG, thoracic cord segments below (T9–T11) and above (T6–T8) the lesion, along with cervical and lumbar enlargements, were rapidly dissected out at 0–4°C, and immediately frozen in liquid nitrogen to be stored at −80°C. In some experiments, spinal cord samples were further sectioned by a medio-vertical cut to separate dorsal and ventral halves. Total RNA was extracted using the NucleoSpin RNA II extraction kit (Macherey-Nagel, 67722 Hoerdt, France) and quantified using NanoDrop. First-stranded cDNA synthesis (from 660 ng total RNA per 20 µL reaction mixture) was carried out using High Capacity cDNA reverse transcription kit (Applied Biosystems, Courtaboeuf, France). PCR amplification, in triplicate for each sample, was performed using ABI Prism 7300 (Applied Biosystems), TaqMan Universal PCR Master Mix No AmpErase UNG (Applied Biosystems) and Assays-on-Demand Gene Expression probes (Applied Biosystems) for targets'genes: *ATF3* (assay ID Rn00563784_m1), *GFAP* (Rn01460868_m1), *OX-42* (Rn00709342_m1), *IL-1β* (Rn00580432_m1), *IL-6* (Rn00561420_m1), *TNF-α* (Rn00562055_m1), *IL-10* (Rn00563409_m1), *BDNF* (Rn02531967_s1), *TLR4* (Rn00569848_m1), *P2×4* (Rn00580949_m1), *P2×7* (Rn00570451_m1). mRNA determinations were made with reference to the reporter gene encoding glyceraldehyde 3-phosphate dehydrogenase (*GaPDH*; Rn99999916_s1). The polymerase activation step at 95°C for 15 min was followed by 40 cycles of 15 s at 95°C and 60 s at 60°C. The validity of the results was checked by running appropriate negative controls (replacement of cDNA by water for PCR amplification; omission of reverse transcriptase for cDNA synthesis). Specific mRNA levels were calculated after normalizing from *GaPDH* mRNA in each sample. Data are presented as relative mRNA units compared to control (sham) values (see [21]).

### Statistical Analyses

All values are expressed as means ± S.E.M. For von Frey filaments tests, the data were analyzed by one-way ANOVA for repeated measures (effect of a drug over time) followed by Dunnett's test. Statistical evaluations of SCT-induced changes in behavioral responses to thermal stimulations were made using the Student's t test. For qRT-PCR data, the 2^−ΔΔCt^ method [26] was used for analysis of the relative changes in specific mRNA levels and for graphic representations (RQ Study Software 1.2 version; Applied Biosystems). For analysis of the time course expression of the targets'genes, a two-way ANOVA was performed, followed by Bonferroni test for comparison of SCT rats versus respective (sham) controls at each time. The critical level of statistical significance was set at P<0.05.

## Results

### Physiological State of Spinal Cord Transected Rats

After full recovery from anaesthesia, SCT rats first showed hindlimb paralysis and flabbiness. Although they moved in their cage without major difficulty and could access food and water as readily as before the surgery, SCT rats stopped gaining weight for the first week after surgery (−6.3±3.8 g, mean ± S.E.M., n = 8), in contrast to sham-operated animals (+43±2 g, mean ± S.E.M., n = 8); but, afterwards, weight gain was parallel in both SCT- and sham-operated rats (+175.4±28.3 g and +171.2±12.5 g from day 7 to day 30 post-surgery, respectively, means ± S.E.M., n = 8 in each group).

Most striking symptoms were urinary retention and/or hematuria. Hematuria disappeared after 3 or 4 days without any specific treatment. To deal with urinary retention, we had to trigger off the miction reflex by rubbing the bladder once a day during 8–9 days on average. Then, the reflex recovered completely. It also happened that some SCT rats had an accelerated gut transit with diarrhea for the first 3 days post surgery. Later on, such gut disorders were only exceptionally observed. On the other hand, SCT rats had their fur a little bit more tousled than sham animals, but it stayed very clean in areas located both rostrally and caudally of the lesion site, most probably through grooming (that we regularly noted) by their cage mate. Abnormal suffering (with signs such as skin scratching) and/or autotomy were never observed when SCT rats were housed two per cage, as always used for these studies.

Immediately after the surgery and during usually 9–10 days, SCT rats showed paraplegia, first characterized by a total absence of reaction when hindlimbs were mechanically stimulated with von Frey filaments exerting pressure up to the cut-off value (100 g) (Fig. 1). This was followed by a hypo-reflexia which progressively vanished up to normal-like response (as in control unoperated rats) to mechanical stimulation which was usually recovered two weeks post surgery. Later on, SCT rats developed a hyper-reflexia with a pressure threshold value to trigger brisk hindpaw withdrawal strikingly lower (−80%) than that determined in sham rats up to at least 7 weeks post-surgery (Fig. 1). All along the observation period, SCT rats had paralyzed hindlimbs with spasticity, rigidity and tonicity. They also had frequent spontaneous movements of the tail and hindlimbs (shaking), and developed uncoordinated flexion and extension movements.

### Development and Localization of Mechanical Allodynia

Among all the body areas tested, only the lesion site on the back and the hindlimbs (see above) showed altered behavioral responses in the von Frey filaments test in SCT- compared to sham-operated rats.

**Figure 2:**
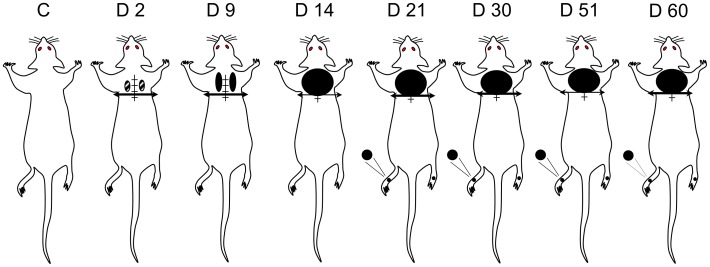
Body territories with increased mechanical sensitivity in spinal cord-transected rats. Pressure threshold values to trigger nocifensive responses were determined using a graded series of von Frey filaments applied throughout the body. Comparison with sham-operated rats (C) showed that pressure threshold values differed in SCT rats only in a limited territory (6 cm^2^) bordering rostrally the spinal cord section (at T8–T9, horizontal bar with arrow heads) and in hindpaws (black areas tested), where reactions were obtained for pressure values significantly less than in controls. Time course (day 2 to day 60) changes in spinal cord transected rats showed that supersensitivity (allodynia) in the at-level area just rostral to the lesion was already detected at day 2 (D2) post-surgery, then extended and increased up to a plateau reached at D14 post-surgery. At hindpaw level, supersensitivity developed much later (from D21 post-surgery). Data were obtained in 8–14 rats at each time.

Within a few days after SCT, supersensitivity to mechanical stimulation appeared at the lesion site. From day 2 to day 9, such supersensitivity was mostly rostro-lateral to the lesion site within small areas on both sides (Fig. 2). Then, the supersensitive territory extended medially and laterally to cover an approximately 6 cm^2^ cutaneous area just rostral to the thoracic cord transection. In contrast, no supersensitivity was detected behind the transection, and, indeed, SCT rats did not react even to a 100 g pressure exerted by von Frey filament applied within the cutaneous territory on the back, caudal to the transection.

**Figure 3:**
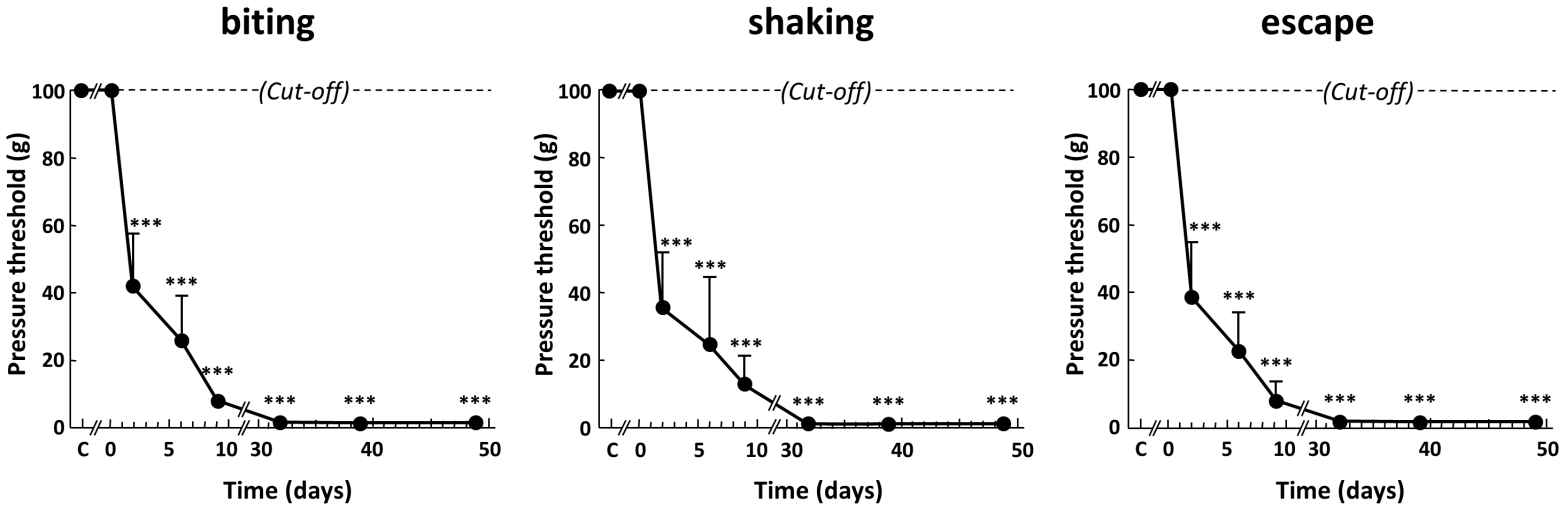
Time-course changes in nocifensive reactions to von Frey filaments application in the « at-level » allodynic territory rostral to the lesion in spinal cord-transected rats. Pressure threshold values to trigger biting (of the filament), shaking or escape were determined using the “up-down” method with a graded series of von Frey filaments applied onto the allodynic at-level area on the back at various times (in days) after surgery (0 on abscissa). Each bar is the mean + S.E.M. of independent determinations in 8 rats. *** P<0.001 compared to control (intact) rats (C on abscissa). One-way ANOVA for repeated measures followed by Dunnett's test.

Further assessment of supersensitivity to application of von Frey filaments within the 6 cm^2^ area just rostral to the lesion led to identify three different aversive reactions: biting, shaking and escape (Fig. 3), in agreement with previous observations in SCI rats [5]. Determinations of pressure threshold values to trigger each of these behaviors showed parallel time-course decreases, down to very low levels that were reached 10–14 days after surgery and remained unchanged for the 7-weeks-observation period (Fig. 3).

### Thermal Sensitivity

**Figure 4:**
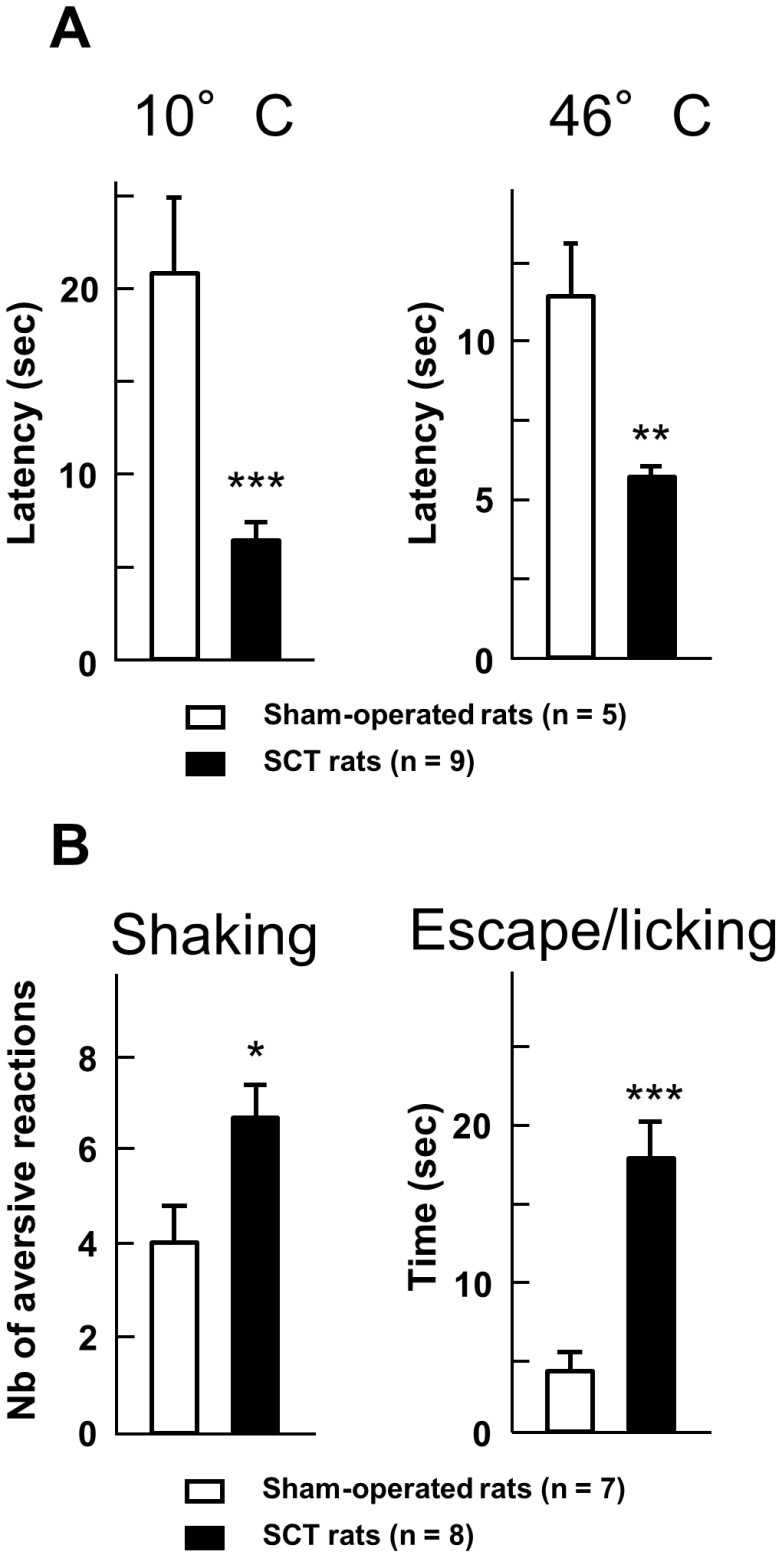
Hyper-responsiveness to thermal stimulation in spinal cord-transected rats. **A**– Latency (in sec) to hindpaw withdrawal was determined after paw immersion into a bath of hot (46°C) or cold (10°C) water, two weeks after the surgery. Each bar is the mean + S.E.M. of independent determinations in 9 SCT rats and 5 sham-operated rats. ** P<0.01, *** P<0.001 compared to respective values in sham-operated rats. Student's t test. **B** – Behavioral responses to the acetone drop test applied at the surgical scar two weeks after surgery. The number of shakes and the time (in sec) spent in escape attempts and licking of the back were measured for one minute after acetone drops application. Each bar is the mean + S.E.M. of independent determinations in 8 SCT rats and 7 sham-operated rats. * P<0.05, ** P<0.01 compared to respective values in sham-operated rats. Student's t test.

To make sure that no bias due to possible changes in skin temperature occurred in SCT- versus sham-operated rats, we first measured hindpaw skin temperature just prior performance of the paw immersion test, two weeks post surgery. Under controlled environmental conditions (with ambient temperature at 22±1°C; see Materials and Methods), hindpaw skin temperature was of 30.1±1.0°C and 29.8±0.5°C (means ± S.E.M. of 8 independent determinations in each group) in SCT- and sham-operated rats, respectively, indicating the lack of incidence of SCT on this parameter. However, clear-cut differences between SCT- and sham-operated rats were noted in withdrawal latencies after hindpaw immersion in cold (10°C) as well as hot (46°C) water. As shown in Figure 4A, SCT rats reacted with much shorter latencies compared to sham-operated animals, as expected of increased sensitivity to both cold and hot stimulation two weeks post SCT.

Further evaluation of cold hypersensitivity was made using the acetone drop test applied at the lesion site, where SCT rats developed mechanical allodynia (Fig. 2). As illustrated in Figure 4B, both the number of trunk shakes and the time spent in back licking and escape attempts for the first min after acetone drops application were significantly increased in SCT- compared to sham-operated animals (+67% and +400%, respectively).

### Pharmacological Studies

#### Effects of Opioïdergic Drugs (Morphine and Tapentadol) on At-Level Mechanical Allodynia

**Figure 5:**
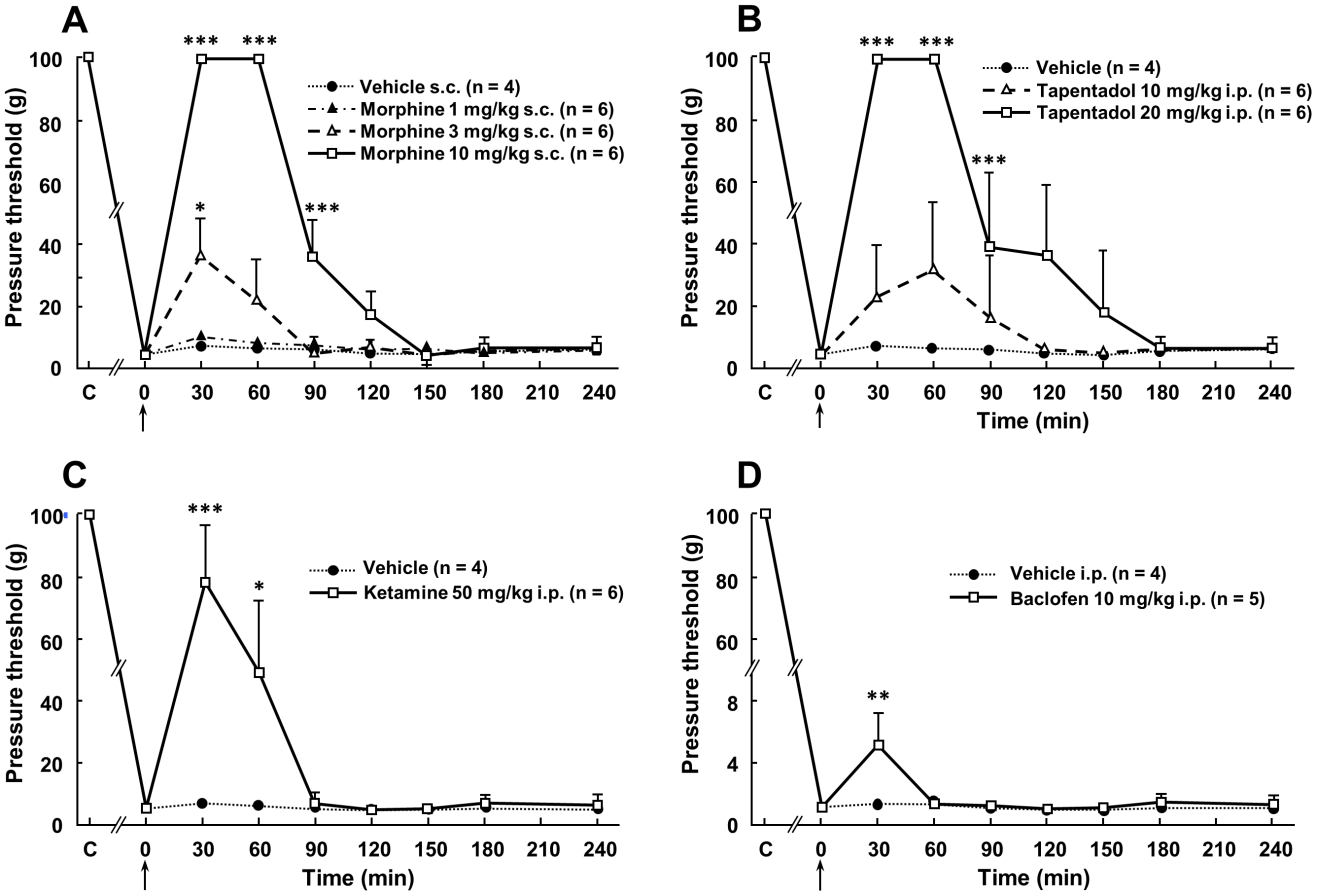
Anti-allodynic effects of acute administration of morphine (A), tapentadol (B), ketamine (C) or baclofen (D) in spinal cord-transected rats. Acute administration of morphine (1, 3 or 10 mg/kg s.c.), tapentadol (10 or 20 mg/kg i.p.), ketamine (50 mg/kg i.p.), baclofen (10 mg/kg i.p.) or their respective vehicle was performed (0 on abscissa, arrow) in rats whose spinal cord had been transected at T8–T9 level one month before. Pressure threshold values to trigger nocifensive biting were determined using von Frey filaments applied within the at-level allodynic territory at various times after treatment. Each point is the mean + S.E.M. of independent determinations in n rats. C on abscissa: Control (naive) rats (prior to surgery). P<0.05, ** P<0.01, *** P<0.001 compared to respective values in vehicle-treated rats. One-way ANOVA for repeated measures followed by Dunnett's test.

As treatments with opioids were shown to reduce pain in humans with spinal cord lesions [27], [28], we investigated whether morphine (1, 3 and 10 mg/kg s.c.) was effective to reduce at-level mechanical allodynia in SCT-rats. Acute treatment was performed 30 days after the surgery, when pressure threshold to elicit biting behavior in response to von Frey filament application had reached its minimum value (Fig. 3). As illustrated in Figure 5A, morphine exerted a dose-dependent effect: it was inactive at 1 mg/kg s.c., but increased pressure threshold value at higher doses, with complete suppression of allodynia-like response 30 and 60 min after administration of the highest dose tested (10 mg/kg s.c.). Confirmation of the anti-allodynic efficacy of opiate receptor activation was made with tapentadol, a mixed mu opioid receptor agonist and noradrenaline reuptake inhibitor with potent antalgic properties [29], which also reversed SCT-induced mechanical allodynia in a dose-dependent manner. As shown in Figure 5B, tapentadol at 10 mg/kg i.p. slightly increased the pressure threshold value, but the dose of 20 mg/kg i.p. completely suppressed allodynia-like response 30 and 60 min after its administration to SCT rats.

#### Effects of Ketamine on At-Level Mechanical Allodynia

Ketamine is well known to reduce pain in humans suffering from spinal cord injury, and its pain alleviating efficacy has also been reported in rat models of SCI, such as the one obtained by spinal cord contusion [30]. In SCT rats, acute administration of ketamine (50 mg/kg i.p.) induced a significant increase in pressure threshold value to trigger nocifensive response to von Frey filament application within the allodynic cutaneous area (Fig. 5C). At its maximum, 30 min after treatment, pressure threshold value reached 77.3±17.6 g (from 0.96 g±0.39 g before treatment, means ± S.E.M. of 6 determinations), which was not significantly different from the cut-off value corresponding to the non-allodynic state (in naïve rats, before surgery). However, this effect vanished rapidly because mechanical allodynia was completely restored 90 min after ketamine administration (Fig. 5C).

#### Effects of Baclofen on At-Level Mechanical Allodynia

Because baclofen, a GABA B receptor agonist, is often prescribed to reduce SCI-induced spasticity in humans, and is endowed with anti-neuropathic pain properties [31], we investigated whether this drug could reduce at-level mechanical allodynia in SCT rats. Indeed, baclofen induced a limited and transient increase (p<0.05) in pressure threshold value, from 0.6±0.4 g before treatment to 5.0±2.1 g 30 min after i.p. administration of this drug at 10 mg/kg (Fig. 5D).

#### Effects of Anticonvulsant Drugs on At-Level Mechanical Allodynia

The calcium channel blockers gabapentin and pregabalin and the benzodiazepine clonazepam are anticonvulsants endowed with anti-neuropathic pain properties both in humans [3], [27] and in rodent models [32], [33], and we tested whether these drugs also exerted anti-allodynic effects in SCT rats. In fact, acute treatments with either gabapentin (30 mg/kg i.p.), pregabalin (30 mg/kg i.p.) or clonazepam (0.25 mg/kg i.p.), at doses devoid of any inhibitory effect on locomotor coordination (as assessed using the rotarod test; not shown), had no significant effect on pressure threshold to trigger nocifensive response in SCT rats (Table 1). Some increase in pressure threshold values was noted with higher doses of clonazepam (2 mg/kg i.p.) and gabapentin (100 and 300 mg/kg i.p.), but rats presented profound ataxia after such treatments (not shown).

#### Effects of Other Drugs on At-Level Mechanical Allodynia

As detailed in Table 1, the antidepressant amitriptyline, alone or combined with gabapentin, the anti-migraine drug naratriptan, the 5-HT_1A/7_ receptor agonist 8-OH-DPAT, the 5-HT_3_ receptor antagonist ondansetron, the BDNF-Trk B receptor blocker cyclotraxin B, at effective doses to reduce pain in validated neuropathic models in rodents [34]–[39], exerted no anti-allodynic effects up to 3 hours after acute administration in SCT rats.

### Neuroinflammatory and Neuroplasticity Markers in Spinal Cord and DRG of SCT rats

#### Spinal Cord

**Figure 6:**
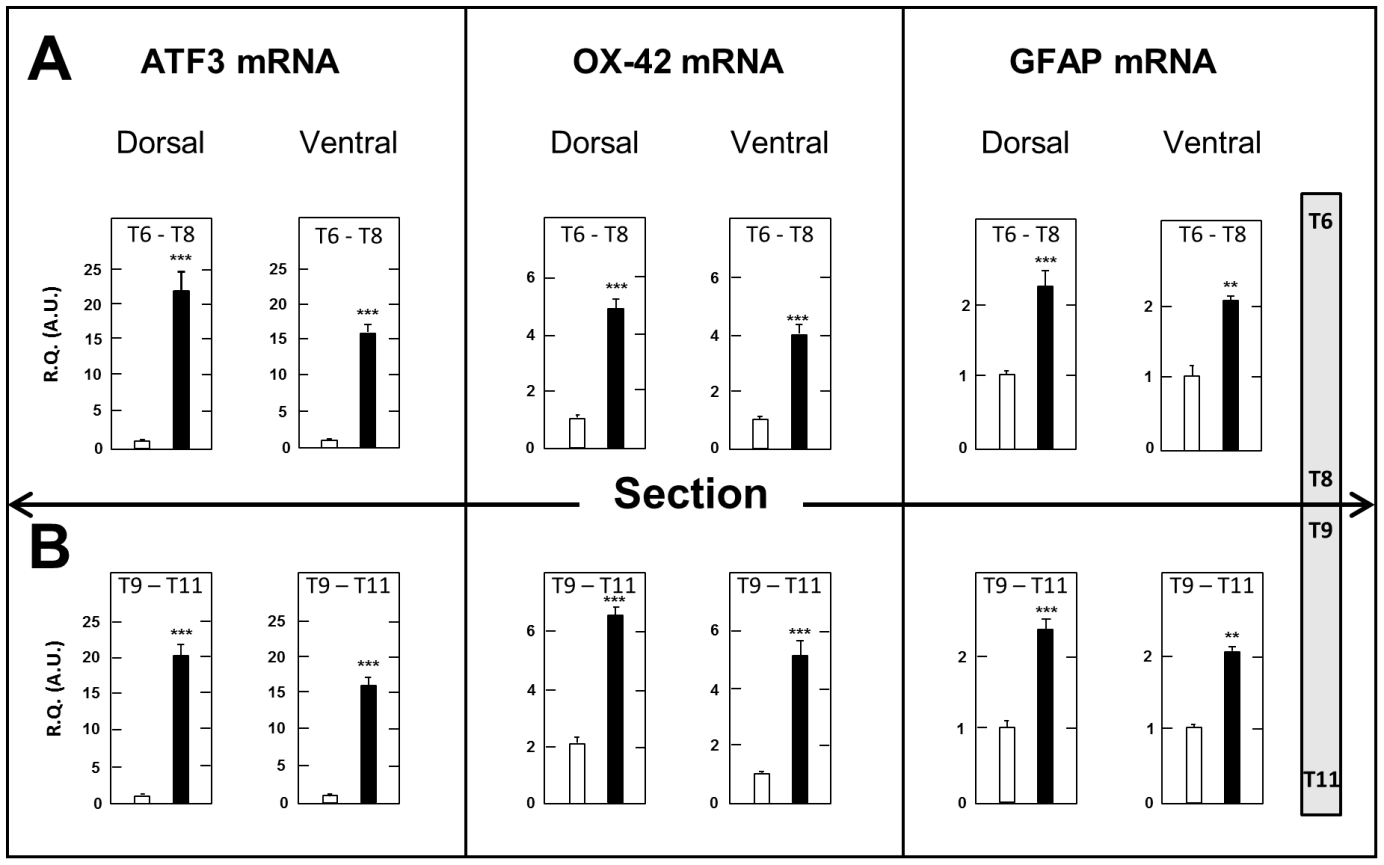
Increased expression of ATF3, OX-42 and GFAP mRNAs in the dorsal and ventral halves of spinal cord segments just above (T6–T8) and below (T9–T11) the surgery level in spinal cord-transected rats. Real time RT-qPCR determinations were made at day 17 after surgery. Data are expressed as the ratio of specific mRNA over GaPDH mRNA [R.Q.(A.U.)]. Each bar is the mean + S.E.M. of 10 independent determinations in both SCT (black bars) and sham-operated (empty bars) rats. *** P<0.001 compared to respective values in sham-operated rats. Two-way ANOVA followed by Bonferroni test.

A first series of determinations consisted of measuring the tissue concentrations of transcripts encoding the neuronal injury marker ATF3, the macrophage-microglial activation marker OX-42 and the astrocytic marker GFAP [21] in the dorsal and ventral halves of spinal cord segments just above (T6–T8) and just below (T9–T11) the surgery level in SCT- compared to sham-operated rats. Measurements were made at day 17 post surgery, when both mechanical (Fig.3) and thermal (Fig.4) allodynia had fully developed. As shown in Figure 6, expression of these three genes was markedly upregulated in both dorsal and ventral halves in segments above and below the section compared to sham-operated rats. Upregulation of ATF3 mRNA was slightly larger in dorsal spinal cord above and below SCT (×20.8- and ×21.1-fold, respectively) than in the corresponding ventral spinal cord segments (×15.7 and ×15.1-fold, respectively). On the other hand, no significant differences were noted between SCT-induced elevation of OX-42 mRNA and GFAP mRNA levels in the dorsal versus the ventral halves of spinal segments above and below SCT. Accordingly, no further distinction between the dorsal and ventral halves was made in subsequent experiments, and whole spinal cord segments were dissected out and processed for investigating the time-course changes in neuroinflammatory and neuroplasticity markers after thoracic cord transection.

**Figure 7:**
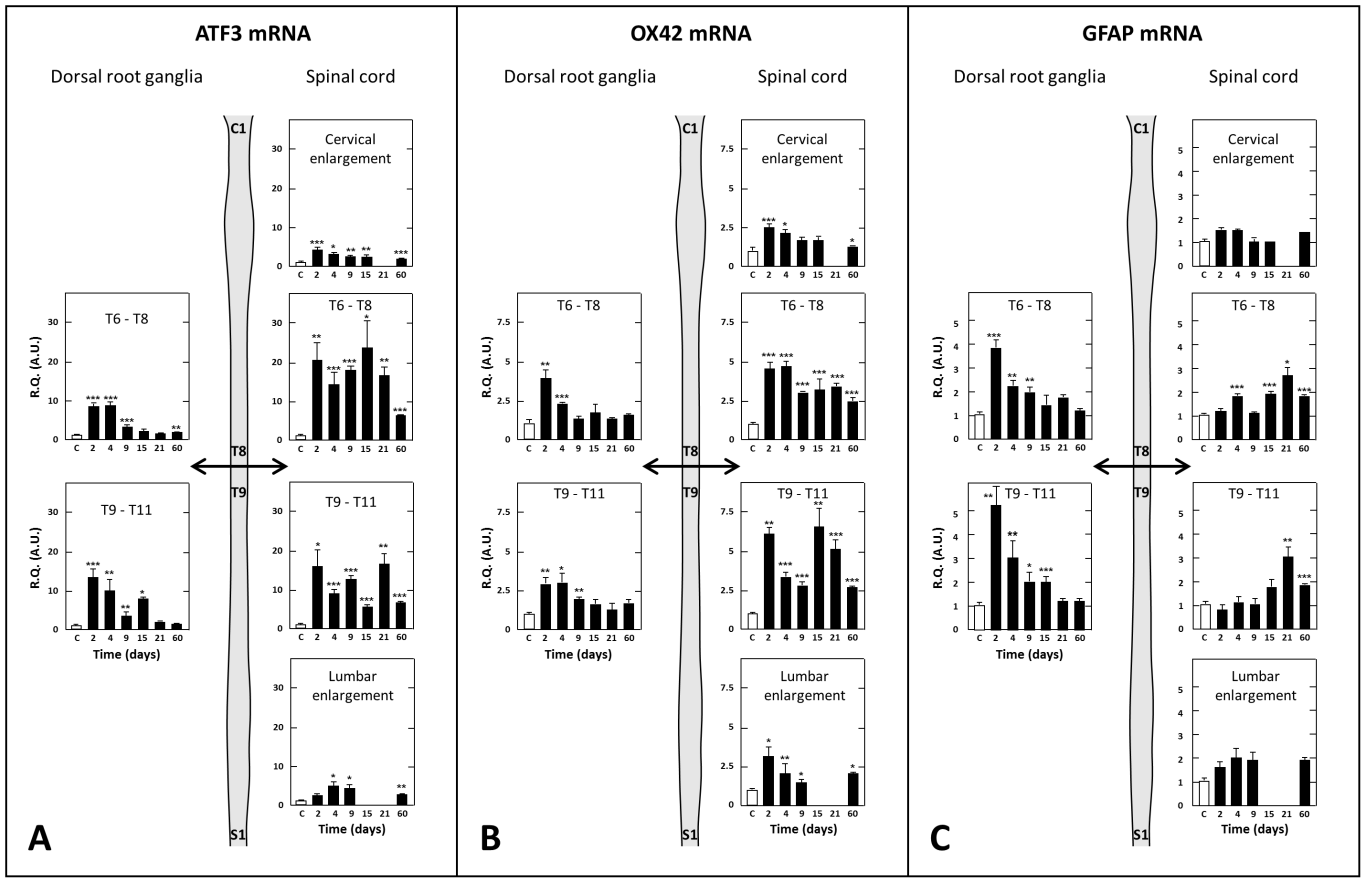
Time-course changes in tissue levels of transcripts encoding ATF3 (A), OX-42 (B) or GFAP (C) in dorsal root ganglia and spinal cord at various times after spinal cord transection. Real-time RT-qPCR determinations were made in T6–T8 and T9–T11 dorsal root ganglia, T6–T8 and T9–T11 spinal cord segments and the cervical and lumbar enlargements at various times (in days, D, abscissa) after spinal cord transection at T8–T9 level. Data are expressed as the ratio of specific mRNA over GaPDH mRNA [R.Q.(A.U.)]. Each bar is the mean + S.E.M. of n independent determinations (D2, D4, D9, D15, D21: n = 6; D60: n = 12). Sham values at every postoperative time are pooled under “C” (control) on abscissa. * P<0.05, * P<0.01, *** P<0.001 compared to respective values in sham-operated rats (C).Two-way ANOVA followed by Bonferroni test.

As shown in Figure 7, already on day 2 post-surgery, ATF3 mRNA levels were 16.0- and 21.0-fold higher in thoracic spinal cord segments just caudal and rostral to the section, respectively, than in corresponding tissues from sham-operated rats. This up-regulation was long lasting as it persisted, but to a lower extent, up to the last observation day (×7.0 and 6.7 on day 60 post-surgery) (Fig. 7A). As illustrated in Figure 7A, a long lasting up regulation of ATF3 mRNA was also detected in both the cervical and lumbar enlargements of the spinal cord in SCT rats. However, this change was of much lower amplitude than in thoracic segments. OX42 mRNA levels were also markedly increased in thoracic segments of the spinal cord just caudal and rostral to the section on day 2 post-surgery (×6.2 and 4.8, respectively), and remained significantly elevated until day 60 (×2.8 and 2.5, respectively) (Fig. 7B). A long lasting up-regulation of OX-42 mRNA was also noted in both the cervical and lumbar enlargements of the spinal cord. However, it was of lower amplitude than in thoracic segments (Fig. 7B). The time course of SCT-induced changes in GFAP mRNA levels differed from those of the former two transcripts, as the observed up-regulation was delayed and relatively less pronounced (×3 at maximum) (Fig. 7C). However, these changes persisted to similar extents up to the last observation day (day 60 post surgery). In cervical and lumbar enlargements, only slight, generally non significant, increases in GFAP mRNA levels were observed in SCT rats, but they were also of long duration (Fig. 7C).

**Figure 8:**
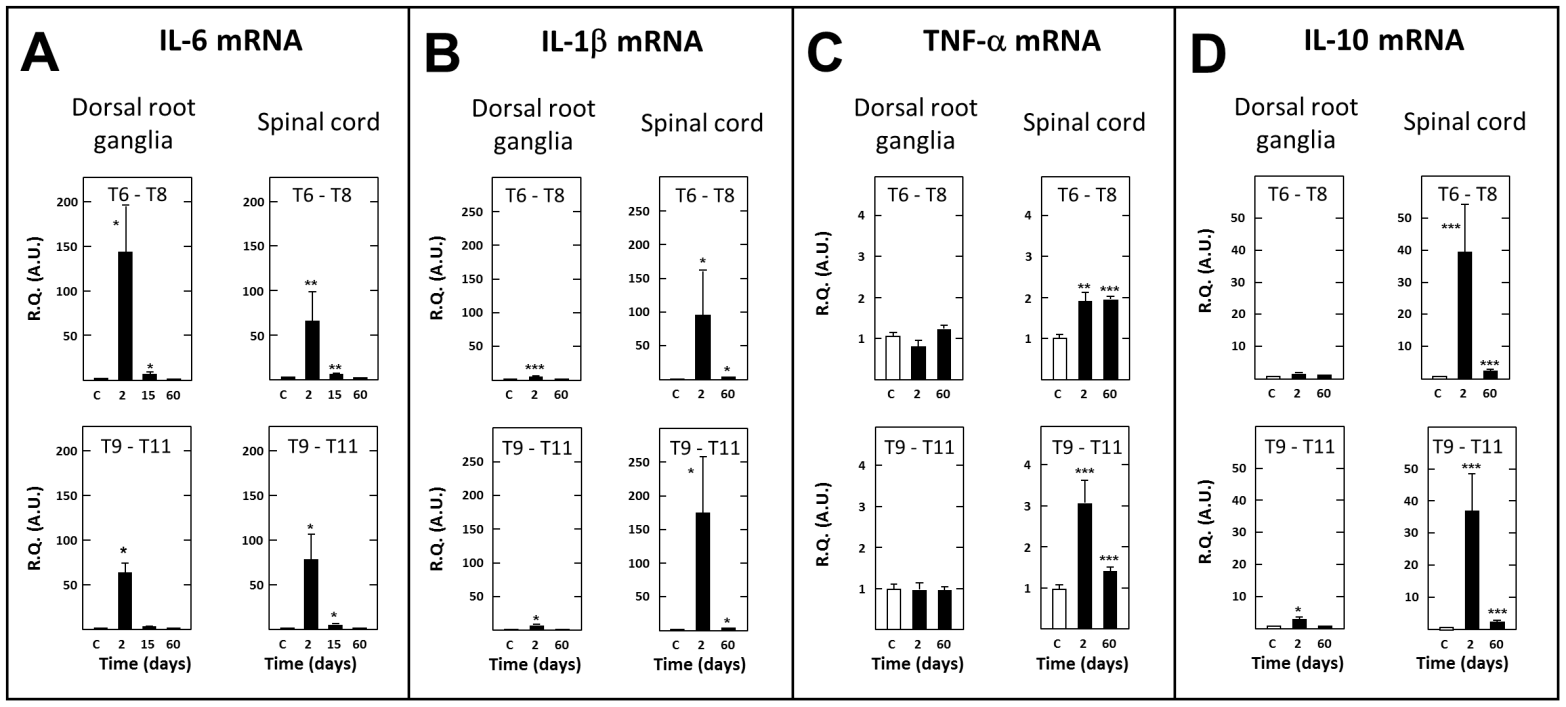
Short- and long-term changes in levels of transcripts encoding IL-6 (A), IL-1β (B), TNF-α (C) and IL-10 in dorsal root ganglia and spinal tissues in spinal cord-transected rats. Real-time RT-qPCR determinations were made in T6–T8 and T9–T11 dorsal root ganglia and T6–T8 and T9–T11 spinal segments at day (D) 2, 15 or 60 (abscissa) after spinal cord transection at T8–T9 level. Data are expressed as the ratio of specific mRNA over GaPDH mRNA [R.Q.(A.U.)]. Each bar is the mean + S.E.M. of n independent determinations (D2, D15: n = 6; D60: n = 12). Sham values at every postoperative time are pooled under “C” (control) on abscissa. * P<0.05, * P<0.01, *** P<0.001 compared to respective values in sham-operated rats (C). Two-way ANOVA followed by Bonferroni test.

Concerning pro-inflammatory cytokines, a massive increase in IL-6 mRNA levels was observed as soon as 2 days after the section in thoracic segments bordering caudally (x 76.8 as compared to sham-operated rats) and rostrally (x 66.4) the section (Fig. 8A). A modest up-regulation was still observed on day 15 but not on day 60 post surgery. In contrast, no significant changes in IL-6 mRNA levels were detected in both the cervical and lumbar enlargements of the spinal cord at any time after SCT as compared to transcript levels measured in the same tissues of sham-operated rats (not shown).

The levels of IL-1β mRNA were also markedly increased 2 days after surgery in thoracic segments bordering caudally (x 172.2 as compared to sham-operated rats) and rostrally (x 98.6) the transection (Fig. 8B). Significant increases in IL-1β mRNA levels still persisted in caudal- and rostral-level segments on day 60 post-surgery, but to a much lower extent than on day 2. Similar but less pronounced changes in TNF-α mRNA levels were noted with a significant up-regulation in thoracic segments on day 2 post surgery (×3.0 caudally and ×1.9 rostrally to the section, respectively) (Fig. 8C). On day 60, a significant increase in TNF-α mRNA levels was still detected principally in thoracic segments rostral to the transection (x 1.9) (Fig. 8C). Finally, tissue concentrations of mRNA encoding the anti-inflammatory cytokine IL-10 were also markedly increased on day 2 after transection in both caudal-level (x 36.3) and rostral-level (x 38.7) thoracic segments, and an up-regulation of much lower amplitude was still detected on day 60 post surgery (Fig. 8D).

**Figure 9:**
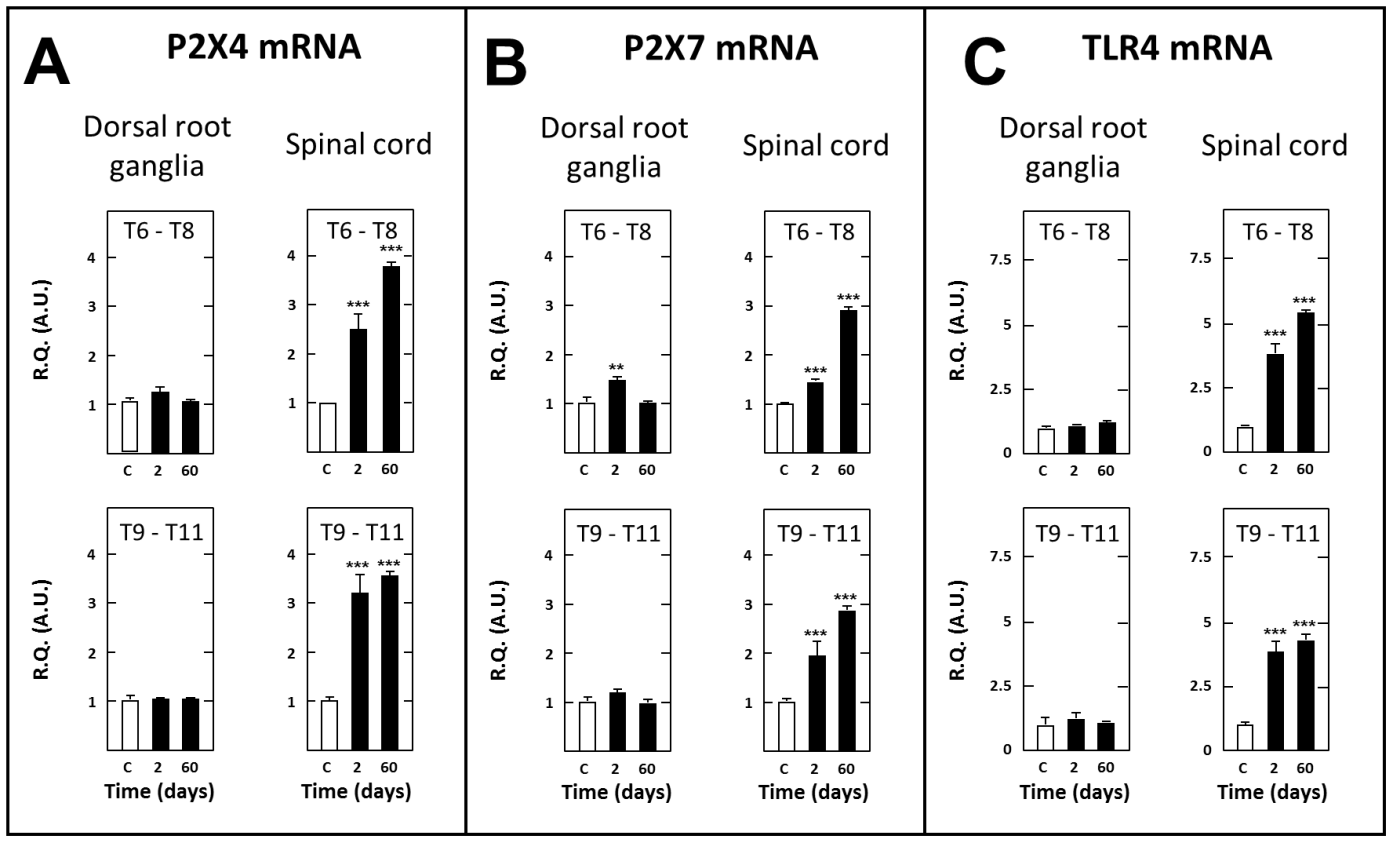
Short- and long-term changes in levels of transcripts encoding P2×4 (A), P2×7 (B) and TLR4 (C) in dorsal root ganglia and spinal tissues in spinal cord-transected rats. Real-time RT-qPCR determinations were made in T6–T8 and T9–T11 dorsal root ganglia and T6–T8 and T9–T11 spinal segments at day 2 or 60 (abscissa) after spinal cord transection at T8–T9 level. Data are expressed as the ratio of specific mRNA over GaPDH mRNA [R.Q.(A.U.)]. Each bar is the mean + S.E.M. of n independent determinations (D2: n = 6; D60: n = 12). Sham values at every postoperative time are pooled under “C” (control) on abscissa. ** P<0.01, *** P<0.001 compared to respective levels in sham-operated rats (C). Two-way ANOVA followed by Bonferroni test.

In contrast with the aforementioned transcripts, BDNF mRNA levels were reduced in spinal cord tissues of SCT rats, both on days 2 (−49% as compared to sham-operated rats, P<0.05) and 60 (−38%, P<0.05) post surgery in thoracic segments caudal to the section and on day 60 (−23%, P≤0.05) post surgery in thoracic segments rostral to the section (not shown). On the other hand, mRNAs encoding P2×4, P2×7 and TLR4 were upregulated in thoracic segments bordering caudally (×3.2, ×1.8 and ×3.8, respectively) and rostrally (×2.6, ×1.5 and ×3.6, respectively) the transection on day 2 post-surgery. This up-regulation was even more pronounced on post-surgery day 60 (×3.6, ×2.9 and ×4.5 caudal to the section, ×3.8, ×2.9 and ×5.6 rostral to the section, respectively) (Fig. 9A, 9B, 9C).

#### Dorsal Root Ganglia

Like that observed at spinal level, ATF3 mRNA was strongly up-regulated in DRG at T9–T11 caudal level as well as T6–T8 rostral level for the first two weeks after thoracic cord transection (Fig. 7A). Then, significant increases persisted up to the last observation day, two months after surgery, but to a lower extent, only in T6–T8 DRG (Fig. 7A). Transcripts encoding OX-42 (macrophages) and GFAP (satellite glial cells) were also markedly up regulated in DRG at spinal cord segments caudal (T9–T11) and rostral (T6–T8) to the transection. However, this effect was transient, especially at rostral level (T6–T8) where significant increases in OX-42 and GFAP transcripts were noted on days 2–4 and up to day 9 post-surgery, respectively. At caudal level (T9–T11), up regulation of these transcripts lasted a few days more, but three weeks post-surgery, both OX-42 and GFAP transcripts no longer differed in thoracic DRG of SCT- versus sham-rats (Figs 7B,7C).

As illustrated in Figure 8A, mRNA encoding IL-6 also showed a dramatic up-regulation (x 65.6) in T9-T11 DRG at day 2 post surgery. Its levels then decreased rapidly, but remained significantly higher than in sham-operated rats up to day 9 post surgery (x 4.4; not shown). Interestingly, up-regulation of IL-6 mRNA was even larger at day 2 (x 145.0) and remained significant for a longer period (up to day 50 post surgery: ×1.6) in rostral level T6–T8 DRG (Fig. 8A, and data not shown). An up regulation of IL-1β mRNA was also noted in thoracic DRG at day 2 post-surgery (but not at day 60) in SCT rats (Fig. 8B), but this change was of much lower amplitude than that noted at spinal level. Also in sharp contrast with that previously noted at spinal level, TNF-α mRNA was not up-regulated in thoracic DRG of SCT rats, neither at day 2 nor at day 60 post-surgery (Fig. 8C). Finally, the levels of mRNA encoding the anti-inflammatory cytokine IL-10 were found to be slightly increased (x 3.1), but only in DRG caudal to the section (T9–T11) on day 2 post surgery, and at a markedly lower extent than in thoracic cord segments (Fig. 8D).

Further transcripts quantifications confirmed the existence of marked differences between DRG and spinal cord tissues. In particular, BDNF mRNA levels were significantly increased in caudal level T9–T11 DRG at both days 2 (×5.6, P<0.01) and 55 (×1.6, P≤0.05) post-surgery, but only at day 2 (×4.3, P<0.01) in rostral level T6–T8 DRG (not shown). On the other hand, mRNAs encoding P2×4, P2×7 and TLR4, which are all expressed by activated macrophages and satellite glial cells [40]–[42], showed no modification of their expression levels in T9–T11 DRG of SCT rats whatever the time after surgery. Similar negative results were noted in T6–T8 DRG except a modest but significant increase in P2×7 mRNA levels observed on day 2 post surgery (Fig. 9A, 9B, 9C).

## Discussion

Spinal cord transection is a model widely used for the study of induced spasticity, hyper-reflexia and subsequent functional and structural plasticity underlying locomotor recovery under the control of the Central Pattern Generator [11]–[13]. Although neuropathic pain concerns a high proportion of SCI patients, only few investigations have been dedicated to alterations in pain signaling mechanisms in rats with complete SCT. Indeed, a large body of data has already been generated from studies in rodents with partial spinal cord lesion, but unavoidable interindividual variations in the severity and extent of lesion constitute serious limitations of such models (see Introduction). These considerations led us to thoroughly characterize the homogeneous model of complete transection of the spinal cord at thoracic level with regard to its possible relevance for studying central neuropathic pain, associated neuroplasticity changes and responses to drugs used to alleviate pain in SCI patients.

### Clinical State of Spinal Cord Transected Rats

Despite complete transection of the spinal cord, rats showed a relatively good physiological state. The lack of micturition reflex and the hematuria, which are commonly encountered in paraplegic patients [43], usually resolved within 9 days post-surgery. Otherwise, their fur was clean, and very probably because they shared their cage with a congener, autotomia never occurred. Although rats lose weight for the first week after surgery, as a consequence of hindlimb muscles atrophy, they subsequently gained weight at the same rate as sham-operated rats, as expected from animals in good health [44].

### Effects of Spinal Cord Transection on Hindlimb Sensitivity

Just after the lesion, hindlimbs no longer responded by a reflex motor reaction to cutaneous mechanical stimulation at high intensity (with the 100 g von Frey filament). Motor reaction then reappeared progressively up to a level corresponding to that found in control (unoperated) animals around the second week post-surgery. A marked hyper-reflexivity subsequently developed, along with spasticity, which reached their maximum approximately 7 weeks post-surgery and were still fully present on the last day (60) of our study. Marked alterations of motor reflexes also occur in humans with complete spinal cord transection, as evidenced by the exacerbated response in the H reflex of hindlimb muscles [45], [46]. Such facilitated reflex responses may be due to α-motoneurons hyperexcitability [47]. Indeed, spinal cord transection causes an up-regulation of constitutively active 5-HT_2C_ receptors expressed by motoneurons, and the reinforcement of their membrane depolarizing influence has been demonstrated to contribute to motoneuron hyperexcitability in lesioned rats [48]. On the other hand, spasticity could also be accounted for by a down regulation of the potassium-chloride cotransporter KCC2 within the lumbar spinal cord below transection [12]. Although spasticity can be painful in humans, and below-level pain exists in patients with extensive spinal cord injury [2], [49], hyper-reflexivity and spasticity at hindlimb level could not be related to pain behavior in SCT rats because completeness of the lesion prevented the nociceptive messages to reach the sensory cortex where they can generate pain sensation.

Along with mechanical hypersensitivity, SCT rats also developed heat and cold hypersensitivity as shown by the reduced latency of hindpaw withdrawal after immersion in water at 46°C or 10°C (Fig.4). Heat hypersensitivity has already been described in mice after spinal cord contusion and transection [50], and cold hypersensitivity at hindpaw level has been well documented in rats with contused spinal cord [51]. Whether or not similar neuroplasticity mechanisms underlay thermal and mechanical hypersensitivity at hindpaw level in SCT rats is a pending question to be addressed in future studies. In particular, because thermal hypersensitivity was evidenced from a motor response (hindpaw withdrawal), it might have also involved – at least in part - some α-motoneuron hyperexcitability as discussed above about SCT-induced mechanical hypersensitivity.

### At-Level Allodynia

Whereas no behavioral reaction to the application of von Frey filaments within the trunk caudal to the lesion could be elicited in SCT rats, at-level allodynia-like reactions appeared relatively rapidly and reached a maximum 2–3 weeks after surgery. In particular, biting, which is considered as a brainstem response, and escape as a cortical response, were very probably associated with pain in SCT rats [5]. Since sham-operated rats did not develop such behaviors, we can exclude that they might have corresponded to musculoskeletal pain. Instead, at-level mechanical allodynia pain was very probably caused by spinal cord injury itself, as expected of neuropathic pain of central (spinal) origin [2]. Interestingly, 100% of SCT rats developed at-level allodynia, contrary to humans with spinal cord lesion and rats with spinal cord contusion as only a fraction of lesioned subjects suffer from such pain symptoms. Indeed, the prevalence for the rat/human to develop at-level pain depends on the extent of the lesion [52]. Such homogeneous data in SCT rats support the idea that the SCT model might be especially useful to assess the potential effects of drugs aimed at reducing centrally-evoked neuropathic pain and to investigate underlying physiopathological mechanisms.

Even though at-level cold allodynia is frequently seen in SCI patients [53], only few studies have reported this symptom in spinal cord lesioned rodents [5], [54]. Indeed, according to Baastrup et al. [5], only 3% of the rats with contusion of the spinal cord exhibit clear-cut cold allodynia. In contrast, in our study, 100% of SCT rats presented at-level cold allodynia further emphasizing the usefulness of this model for improving experimental group homogeneity. A potential at-level heat allodynia could not be assessed in our studies because of the unavailability of appropriate equipment. Nevertheless, it can be recalled that using a Peltier device, Gao et al. [54] were unable to detect any heat allodynia in spinal cord contused rats.

### Pharmacological Sensitivity of At-Level Mechanical Allodynia in SCT Rats

Only a few drugs among those tested were found to efficiently reduce at-level allodynia when injected acutely in SCT rats. The efficacy of morphine and tapentadol was probably underlain by the capacity of mu opioid receptor activation to inhibit the activity of wide dynamic range neurons in the dorsal horn of the spinal cord [55]. Interestingly, tapentadol had a somewhat more prolonged effect than morphine, may be because of its additional capacity to inhibit noradrenaline reuptake as this monoamine has been shown to be implicated in descending inhibitory control of neuropathic pain [56].

Ketamine also reversed at-level allodynia in SCT rats, in consistence with human data that demonstrated that this NMDA receptor antagonist is especially efficient to reduce allodynia in SCI patients [57]. This marked effect of ketamine, that may be sustained by a temporary inhibition of astrocyte activation, further supports the key role played by glutamate receptors, particularly NMDA receptors, in physiopathological mechanisms underlying neuropathic pain [58].

Finally, the last drug of the series tested which was found to exert some (but modest) anti-allodynic effects in SCT rats was the GABA B receptor agonist, baclofen, commonly used to suppress spasticity in spinal cord injured patients [59]. Spinal cord injury is known to be associated with a decreased tone of inhibitory GABAergic neurotransmission [7], and it can be proposed that baclofen transiently compensated for this deficit, thereby reducing allodynia in SCT rats. In contrast, clonazepam, which is used to alleviate SCI patients from neuropathic pain [27], was inefficient suggesting that GABA A receptor activation was ineffective to inhibit at-level allodynia in SCT rats.

Serotonin is known to play a major role in pain control via the activation of several receptor types [36]. Thus, F13640, a potent and selective 5-HT_1A_ receptor agonist, appeared to be especially effective to suppress allodynia in spinal cord lesioned rats [60]. In our hands, the prototypical 5-HT_1A_ receptor agonist, 8-OH-DPAT, did not reduce allodynia in SCT rats. Yet, this molecule is also an agonist at 5-HT_7_ receptors, whose activation can result in effects opposite to that expected from 5-HT_1A_ receptor activation [61]. Further studies with selective 5-HT_1A_ and 5-HT_7_ receptor ligands have therefore to be performed in order to reach a clear-cut conclusion regarding the potential modulations of at-level allodynia by serotonin acting at these receptors.

Because allodynia-like sensory dysfunctions are associated with migraine [62], we also investigated whether the anti-migraine drug, naratriptan, with potent 5-HT_1B/1D_ receptor agonist properties [36], could alleviate at-level allodynia in SCT rats. Indeed, no effect was observed, possibly because triptans were found to selectively reduce neuropathic pain at cephalic level but not in extra-cephalic territories [35]. Finally, the last 5-HT receptor that we selected for our pharmacological investigations was the 5-HT_3_ type whose implication in modulatory controls of neuropathic pain has been firmly established [63]. In contrast to the capacity of i.t. injection of ondansetron to attenuate neuropathic pain caused by spinal cord compression [64], this treatment was inactive in SCT rats, probably because complete transection of the spinal cord had suppressed the bulbo-spinal connections involved in 5-HT_3_ receptor-mediated effects [38].

Under our acute treatment conditions, neither the antidepressant amitriptyline nor the anticonvulsants gabapentin and pregabalin, which are commonly used to reduce neuropathic pain in SCI patients [3], exerted any significant anti-allodynic effect in SCT rats (Table 1). Indeed, numerous studies showed that these drugs are effective only under chronic treatment conditions [39], [65], and further experiments consisting of repeated administrations of antidepressants and anticonvulsants have to be performed before concluding about their effectiveness or ineffectiveness in the SCT rat model.

Finally, because BDNF and its receptor TrkB play key roles in physiopathological mechanisms underlying neuropathic pain [66], [67], we investigated whether acute TrkB blockade by cyclotraxin B could affect allodynia in SCT rats. Indeed, Constandil et al. [34] reported that this drug can prevent and reverse neuropathic pain caused by peripheral nerve ligation in rats. In contrast, we found that cyclotraxin B was unable to reduce allodynia in SCT rats (Table 1), in line with RT-qPCR determinations which suggested that spinal BDNF expression would not be upregulated (in contrast to that observed in peripheral neuropathic pain models [66], [67]) but rather downregulated after thoracic cord transection, as previously reported after other types of SCI in rats [68], [69].

### Neuroinflammation and Glial Activation in SCT Rats

The transcription factor ATF3 is induced when neurons are injured, and implicated in regeneration and plasticity [21]. Its role in the *maintenance* of central neuropathic pain is the matter of controversy, as it is no longer expressed when pain is still present after spinal cord injury [70]. However, ATF3 implication in the *induction* of central neuropathic pain is supported by data showing that it promotes the expression of the microglial/macrophage marker OX-42 and the astrocyte/satellite glial cell marker GFAP [71], [72], two factors closely associated with neural lesion-evoked neuropathic pain [21], [70], [73]–[75]. Because ATF3 activation is triggered by cellular damages, and this transcription factor is able to repress its own promoter [71], the long lasting up-regulation of ATF3 transcript that occurred after SCT might reflect an ongoing neuronal damage associated with microglia activation. Convergent data in the literature showed that microglia activation is mediated, among others, by purinergic receptors [76] and Toll-Like Receptors [77]. Consistently, we observed, in thoracic cord segments just caudal (T9–T11) and rostral (T6–T8) to the transection, a long lasting (up to 60 days post-surgery) increase in the expression of mRNAs encoding P2XA, P2×7 and TLR4 receptors.

Numerous reports in the literature ascribe to activated microglia an important role in neuropathic pain consecutive to spinal cord injury [70], [73], [78], and the marked induction of OX42 mRNA in SCT rats is congruent with these data. In fact, IL-6, IL-1β, and TNF-α cytokines released from activated microglia can induce, by themselves, central (spinal) sensitization, thus maintaining neuropathic pain [79], [80]. The huge induction of IL-6 and IL-1β that occurred on day 2 post-surgery suggests that these cytokines were involved more in the *induction* than in the *maintenance* of SCT-evoked neuropathic pain. In contrast, TNF-α would be more concerned by pain *maintenance* as SCT-induced up-regulation of its transcript in spinal T6–T8 segments was as pronounced at day 60 as at day 2 post-surgery. The strong increase in IL-10 mRNA that occurred shortly after the lesion might be linked to some inhibitory control of neuropathic pain for the first days after SCT, through the anti-inflammatory potency of this cytokine [81] and/or its neuroprotective effects in spinal cord injured models [82]. Overall, in contrast to that found in spinal tissues, none of the 11 genes studied were up-regulated beyond two weeks post-surgery in DRG above the lesion, supporting the idea that SCT-induced long lasting at-level allodynia did not involve some peripheral hypersensitivity but corresponded mainly, if not exclusively, to central neuropathic pain. Indeed, the short lasting induction of ATF3, OX-42, GFAP and cytokines encoding genes in DRG might have reflected some limited lesion of T8–T9 dorsal roots possibly occurring during surgery for thoracic cord transection. As a matter of fact, it has to be emphasized that our RT-qPCR determinations of time-course changes in mRNA levels will have to be completed by measurements of corresponding proteins in order to validate the inferences made above about the respective implications of pro-inflammatory cytokines and other neuroinflammatory markers in neuropathic pain-inducing mechanisms in SCT rats.

Within the spinal cord, GFAP mRNA up-regulation after SCT was delayed compared to that of transcripts encoding the pro-inflammatory cytokines IL-1β, IL-6 and TNF-α, in line with the idea that early production and release of these cytokines from microglial activation [83] leads to secondary induction of astrogliosis after injury [84]. That astrogliosis with an up-regulation of GFAP [74] - like that found in SCT rats - contributes to neuropathic pain after spinal cord injury is supported by the fact that pharmacological blockade of astroglia activation reduced pain in spinal cord-lesioned rats [73], [85].

## Conclusion

Spinal cord transection at thoracic level in rats appeared to generate a highly reproducible model of at-level neuropathic pain, mainly of central origin, suitable for pharmacological studies aimed at testing innovative treatments targeted specifically on spinal lesion-evoked neuropathic pain. Time course changes in mRNA levels of neuroinflammatory markers induced by the lesion supported the idea that both activated microglia and activated astroglia contributed to neuropathic pain in spinally transected rats. However, further investigations of these markers have to be made at protein level in order to determine more precisely the respective roles of both cell types in mechanisms underlying central allodynia in SCT rats.
