# Correction: Spinal Cord Transection-Induced Allodynia in Rats - Behavioral, Physiopathological and Pharmacological Characterization

**DOI:** 10.1371/journal.pone.0117868

**Published:** 2015-03-17

**Authors:** The PLOS ONE Staff

There is an error in the fourth to last sentence of the Abstract. The correct sentence is: A marked up-regulation of mRNAs encoding ATF3 (neuronal injury) and glial activation markers (OX-42, GFAP, P2X4, P2X7, TLR4) was observed in spinal cord and/or dorsal root ganglia at T6-T11 levels from day 2 up to day 60 post surgery.

There is an error in the sixth sentence of the “Real Time Quantitative RT-PCR Measurements” subsection of the Materials and Methods. The correct sentence is: PCR amplification, in triplicate for each sample, was performed using ABI Prism 7300 (Applied Biosystems), TaqMan Universal PCR Master Mix No AmpErase UNG (Applied Biosystems) and Assays-on-Demand Gene Expression probes (Applied Biosystems) for targets’genes: *ATF3* (assay ID Rn00563784_m1), *GFAP* (Rn01460868_m1), *OX-42* (Rn00709342_m1), *IL-1β* (Rn00580432_m1), *IL-6* (Rn00561420_m1), *TNF-α* (Rn00562055_m1), *IL-10* (Rn00563409_m1), *BDNF* (Rn02531967_s1), *TLR4* (Rn00569848_m1), *P2X4* (Rn00580949_m1), *P2X7* (Rn00570451_m1).

There is an error in the last sentence of the first paragraph of the “Neuroinflammation and Glial Activation in SCT Rats” subsection of the Discussion. The correct sentence is: Consistently, we observed, in thoracic cord segments just caudal (T9–T11) and rostral (T6–T8) to the transection, a long lasting (up to 60 days post-surgery) increase in the expression of mRNAs encoding P2XA, P2X7 and TLR4 receptors.

There is an error in reference 49. The correct reference is: Werhagen L, Budh CN, Hultling C, Molander C (2004) Neuropathic pain after traumatic spinal cord injury—relations to gender, spinal level, completeness, and age at the time of injury. Spinal Cord 42: 665–673.

There is an error in reference 76. The correct reference is: Marcillo A, Frydel B, Bramlett HM, Dietrich WD (2012) A reassessment of P2X7 receptor inhibition as a neuroprotective strategy in rat models of contusion injury. Exp Neurol 233: 687–692.

There is an error in the legend for Fig. 4. Please see the complete, corrected Fig. 4 here.

**Fig 4:**
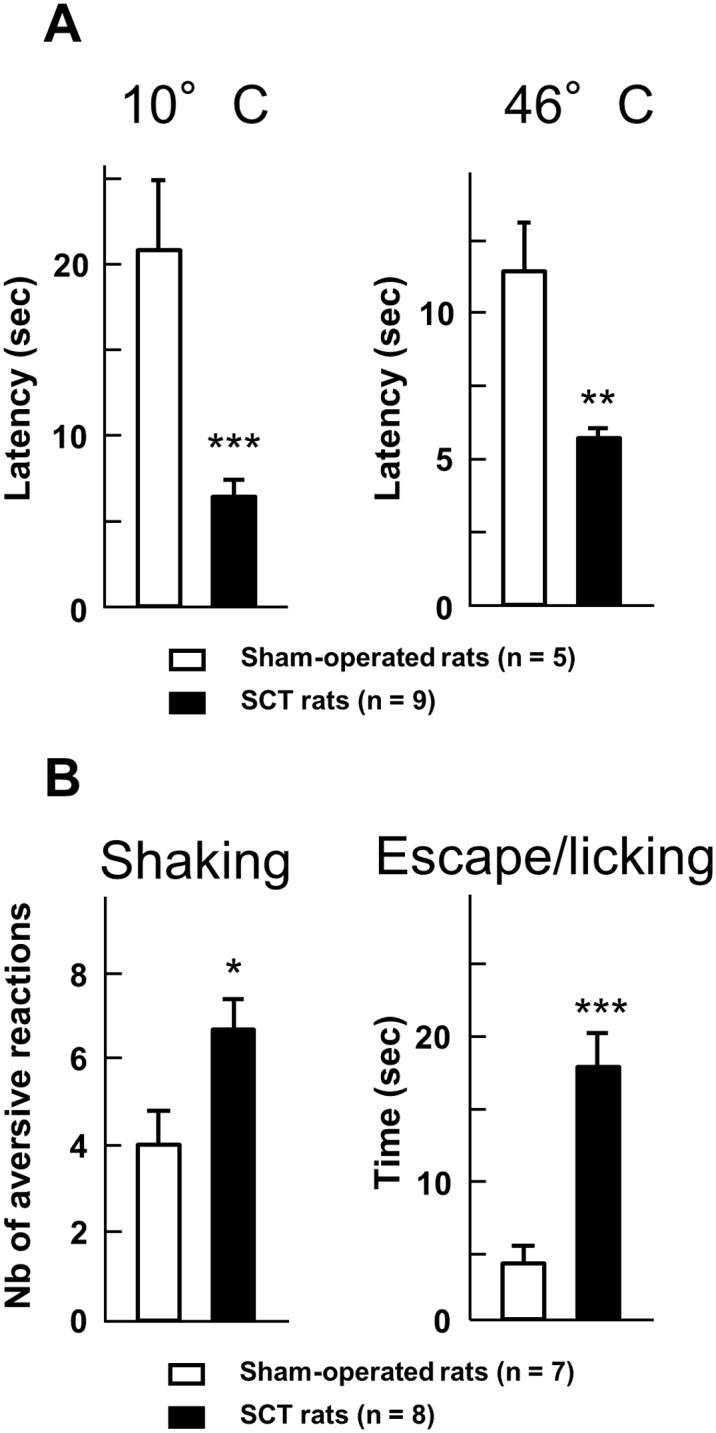
Hyper-responsiveness to thermal stimulation in spinal cord-transected rats. A– Latency (in sec) to hindpaw withdrawal was determined after paw immersion into a bath of hot (46°C) or cold (10°C) water, two weeks after the surgery. Each bar is the mean + S.E.M. of independent determinations in 9 SCT rats and 5 sham-operated rats. ** P<0.01, *** P<0.001 compared to respective values in sham-operated rats. Student’s t test. B – Behavioral responses to the acetone drop test applied at the surgical scar two weeks after surgery. The number of shakes and the time (in sec) spent in escape attempts and licking of the back were measured for one minute after acetone drops application. Each bar is the mean + S.E.M. of independent determinations in 8 SCT rats and 7 sham-operated rats. *P<0.05, ** P<0.01, ***P< 0.001 compared to respective values in sham-operated rats. Student’s t test.

There is an error in the legend for Fig. 5. Please see the complete, corrected Fig. 5 here.

**Fig 5:**
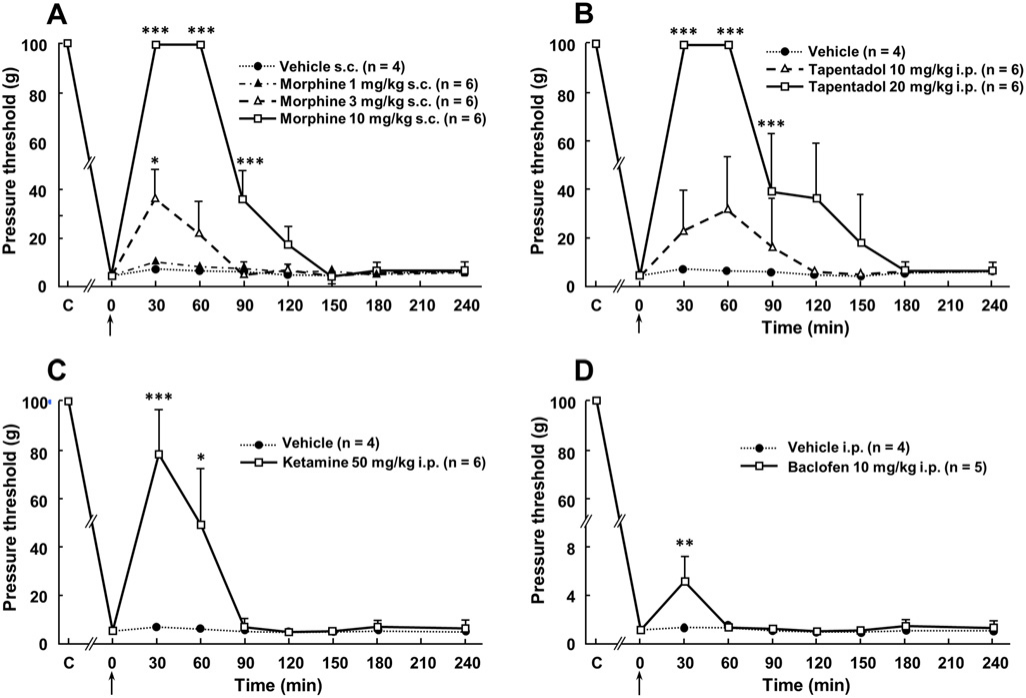
Anti-allodynic effects of acute administration of morphine (A), tapentadol (B), ketamine (C) or baclofen (D) in spinal cord-transected rats. Acute administration of morphine (1, 3 or 10 mg/kg s.c.), tapentadol (10 or 20 mg/kg i.p.), ketamine (50 mg/kg i.p.), baclofen (10 mg/kg i.p.) or their respective vehicle was performed (0 on abscissa, arrow) in rats whose spinal cord had been transected at T8–T9 level one month before. Pressure threshold values to trigger nocifensive biting were determined using von Frey filaments applied within the at-level allodynic territory at various times after treatment. Each point is the mean + S.E.M. of independent determinations in n rats. C on abscissa: Control (naive) rats (prior to surgery). *P<0.05, ** P<0.01, *** P<0.001 compared to respective values in vehicle-treated rats. One-way ANOVA for repeated measures followed by Dunnett’s test.

There is an error in the legend for Fig. 6. Please see the complete, corrected Fig. 6 here.

**Fig 6:**
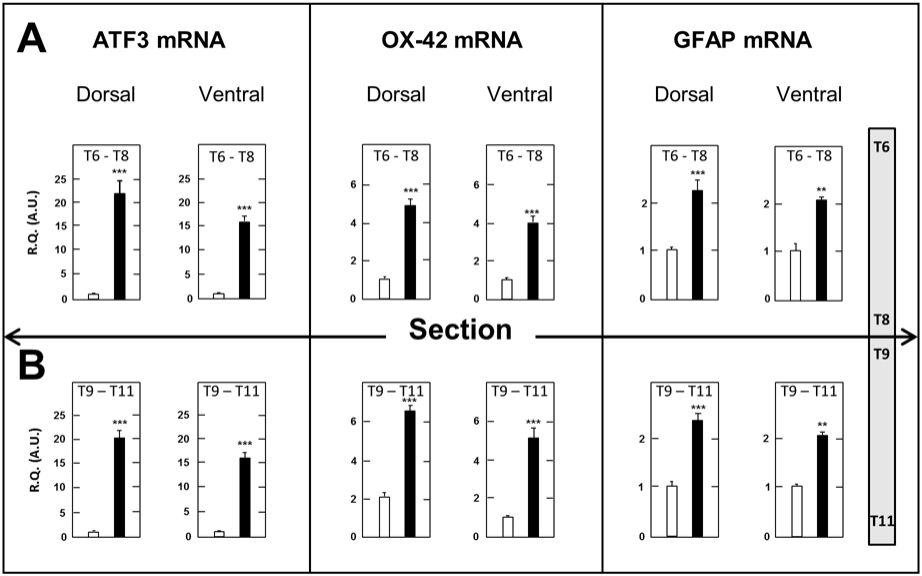
Increased expression of ATF3, OX-42 and GFAP mRNAs in the dorsal and ventral halves of spinal cord segments just above (T6–T8) and below (T9–T11) the surgery level in spinal cord-transected rats. Real time RT-qPCR determinations were made at day 17 after surgery. Data are expressed as the ratio of specific mRNA over GaPDH mRNA [R.Q.(A.U.)]. Each bar is the mean + S.E.M. of 10 independent determinations in both SCT (black bars) and sham-operated (empty bars) rats. **P < 0.01, *** P<0.001 compared to respective values in sham-operated rats. Two-way ANOVA followed by Bonferroni test.

There is an error in the legend for Fig. 7. Please see the complete, corrected Fig. 7 here.

**Fig 7:**
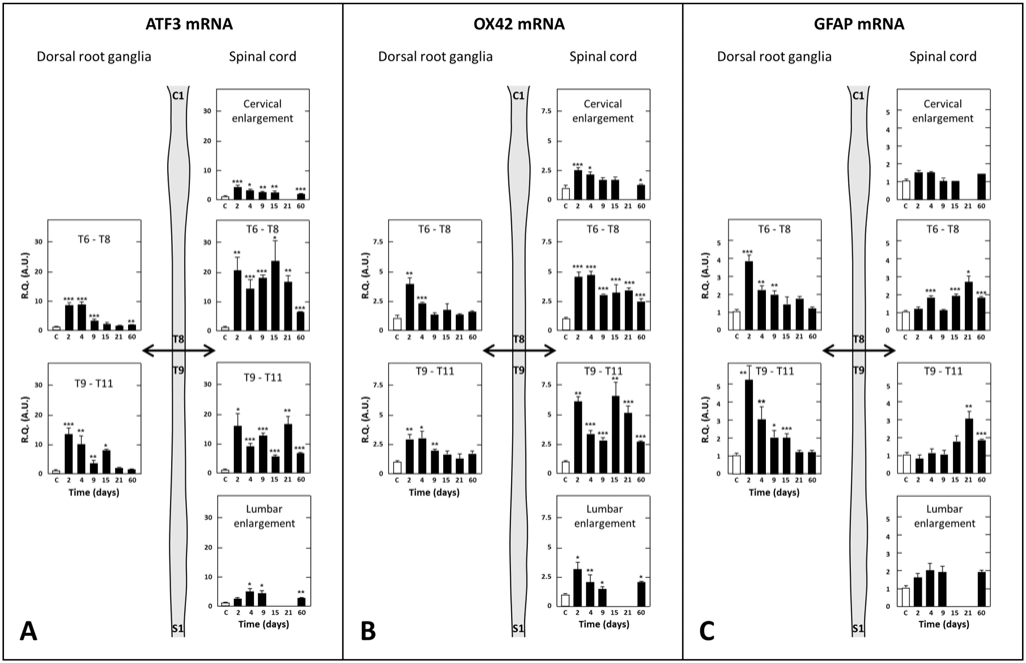
Time-course changes in tissue levels of transcripts encoding ATF3 (A), OX-42 (B) or GFAP (C) in dorsal root ganglia and spinal cord at various times after spinal cord transection. Real-time RT-qPCR determinations were made in T6–T8 and T9–T11 dorsal root ganglia, T6–T8 and T9–T11 spinal cord segments and the cervical and lumbar enlargements at various times (in days, D, abscissa) after spinal cord transection at T8–T9 level. Data are expressed as the ratio of specific mRNA over GaPDH mRNA [R.Q.(A.U.)]. Each bar is the mean + S.E.M. of n independent determinations (D2, D4, D9, D15, D21: n = 6; D60: n = 12). Sham values at every postoperative time are pooled under “C” (control) on abscissa. * P<0.05, **P<0.01, *** P<0.001 compared to respective values in sham-operated rats (C).Two-way ANOVA followed by Bonferroni test.

There is an error in the legend for Fig. 8. Please see the complete, corrected Fig. 8 here.

**Fig 8:**
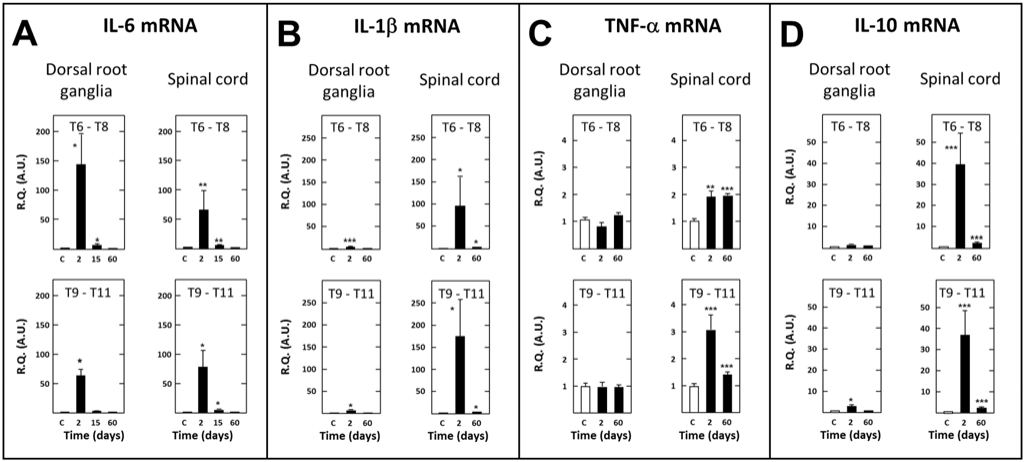
Short- and long-term changes in levels of transcripts encoding IL-6 (A), IL-1β (B), TNF-α (C) and IL-10 in dorsal root ganglia and spinal tissues in spinal cord-transected rats. Real-time RT-qPCR determinations were made in T6–T8 and T9–T11 dorsal root ganglia and T6–T8 and T9–T11 spinal segments at day (D) 2, 15 or 60 (abscissa) after spinal cord transection at T8–T9 level. Data are expressed as the ratio of specific mRNA over GaPDH mRNA [R.Q.(A.U.)]. Each bar is the mean + S.E.M. of n independent determinations (D2, D15: n = 6; D60: n = 12). Sham values at every postoperative time are pooled under “C” (control) on abscissa. * P<0.05, **P<0.01, *** P<0.001 compared to respective values in sham-operated rats (C). Two-way ANOVA followed by Bonferroni test.

There is an error in the legend for Fig. 9. Please see the complete, corrected Fig. 9 here.

**Fig 9:**
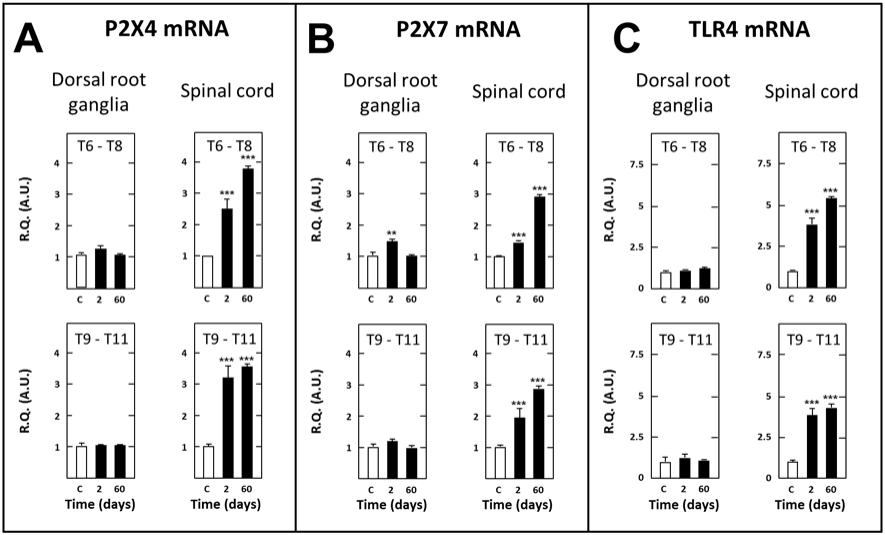
Short- and long-term changes in levels of transcripts encoding P2X4 (A), P2X7 (B) and TLR4 (C) in dorsal root ganglia and spinal tissues in spinal cord-transected rats. Real-time RT-qPCR determinations were made in T6–T8 and T9–T11 dorsal root ganglia and T6–T8 and T9–T11 spinal segments at day 2 or 60 (abscissa) after spinal cord transection at T8–T9 level. Data are expressed as the ratio of specific mRNA over GaPDH mRNA [R.Q.(A.U.)]. Each bar is the mean + S.E.M. of n independent determinations (D2: n = 6; D60: n = 12). Sham values at every postoperative time are pooled under “C” (control) on abscissa. ** P<0.01, *** P<0.001 compared to respective levels in sham-operated rats (C). Two-way ANOVA followed by Bonferroni test.
